# Salivary MicroRNA for Diagnosis of Cancer and Systemic Diseases: A Systematic Review

**DOI:** 10.3390/ijms21030907

**Published:** 2020-01-30

**Authors:** Giacomo Setti, Margherita E. Pezzi, Maria Vittoria Viani, Thelma A. Pertinhez, Diana Cassi, Cristina Magnoni, Pierantonio Bellini, Antonino Musolino, Paolo Vescovi, Marco Meleti

**Affiliations:** 1 Molecular Medicine Ph.D. School, Department of Medicine and Surgery, University of Parma, 43125 Parma, Italy; 2 Dentistry and Oral and Maxillofacial Surgery—Department of Surgical, Medical, Dental and Morphological Science with interest in Transplant Oncological and Regenerative Medicine—University of Modena and Reggio Emilia, Largo del Pozzo 71, 41125 Modena, Italy; pierantonio.bellini@unimore.it; 3 Centro Universitario di Odontoiatria—University of Parma, Via Gramsci 14, 43126 Parma, Italy; margherita.pezzi@gmail.com (M.E.P.); mariavittoriaviani@gmail.com (M.V.V.); diana.cassi@unipr.it (D.C.); paolo.vescovi@unipr.it (P.V.); marco.meleti@unipr.it (M.M.); 4 Department of Medicine and Surgery—Via Volturno 39, 43125 Parma, Italy; thelma.pertinhez@unipr.it; 5 Transfusion Medicine Unit, Azienda USL—IRCCS di Reggio Emilia—Viale Umberto I, 50, 42123 Reggio Emilia, Italy; 6 Dermatology—Department of Surgical, Medical, Dental and Morphological Science with interest in Transplant Oncological and Regenerative Medicine—University of Modena and Reggio Emilia, Largo del Pozzo 71, 41125 Modena, Italy; magnoni.cristina@gmail.com; 7 Medical Oncology and Breast Unit, University Hospital of Parma – Via Gramsci 14, 43125 Parma, Italy; amusolino@ao.pr.it

**Keywords:** saliva, biomarkers, miRNA, cancer, diagnosis

## Abstract

Background: The aberrant expression of microRNAs (miRNAs) has been associated with several diseases, including cancer, inflammatory, and autoimmune conditions. Interest in salivary miRNAs as non-invasive tools for the diagnosis of malignancies and systemic diseases is rapidly increasing. The present systematic review was developed for answering the question: “Are salivary microRNAs reliable biomarkers for diagnosis of cancer and systemic diseases?” Methods: The application of inclusion and exclusion criteria led to the selection of 11 papers. Critical appraisals and quality assessments of the selected studies were performed through the National Institute of Health “Study Quality Assessment Tool” and the classification of the Oxford Center for Evidence-Based Medicine. Results: Seven studies reported statistically significant correlations between one or more salivary miRNAs and the investigated disease. The critical analysis allowed us to classify only two studies (18.2%) as having “good” quality, the rest being scored as “intermediate” (8; 73%) and “poor” (1; 9%). Evidence exists that salivary miR-940 and miR-3679-5p are reliable markers for pancreatic cancer and that miR140-5p and miR301a are promising molecules for the salivary diagnosis of gastric cancer. Conclusions: Further studies, possibly avoiding the risk of bias highlighted here, are necessary to consolidate these findings and to identify new reliable salivary biomarkers.

## 1. Introduction

MicroRNAs (miRNAs or miRs) are short non-coding RNA molecules (about 19–23 nucleotides long) that are involved in the regulation of gene expression at transcriptional and post-translational levels. miRNAs constitute one of the most abundant classes of gene-regulatory molecules. They are consistently part of the transcriptome and their biological influence is widespread; over 30% of the genome is predicted to be actively regulated by miRNAs, and it has been shown that they are involved in the regulation of gene expression and in a variety of cell processes such as apoptosis, proliferation, and differentiation [1].

Different mechanisms have been established as responsible for the miRNAs’ deregulation in systemic disease and cancer. Genetic alterations such as chromosomal rearrangements, genomic amplifications, deletions, or point mutations presumptively play an important role in disease initiation and progression through the aberrant expression of miRNAs located in the affected regions and, subsequently, by deregulation of their downstream mRNA targets. Gene regulation and gene expression are obtained through miRNA binding to the 3′-UTR (untranslated region) of mRNAs and the consequent degradation or translational repression of targeted gene transcripts [2].

The aberrant expression of miRNAs has been associated with a growing number of disease states, including cancer, inflammatory, and autoimmune diseases [1].

In recent years, interest has raised on miRNAs as potential biomarkers for the diagnosis, prognosis, and evaluation of treatment efficacy of several diseases. Profiling miRNA expression is likely to not only allow the identification of a neoplastic tissue and its histological origin, but also to discriminate between different subtypes of malignant lesions. With regard to inflammation, understanding the role of miRNAs in its regulation might be important for helping the comprehension of the pathogenesis of a broad group of diseases [3].

The possibility of evaluating miRNAs in “liquid biopsy” has made rapid progress since 2008, when circulating miRNAs were detected for the first time in the blood of patients with diffuse large B-cell lymphoma and prostate cancer [4,5].

Circulating miRNAs have subsequently been found in several other body fluids, including urine, saliva, and cerebrospinal fluid. The detection of these small molecules in a surprisingly stable form is due to their association with subcellular vesicle-free particles (usually protein complexes) or to the packaging into micro-vesicles or exosomes [6].

Saliva has a very complex composition, including enzymes, antibodies, hormones, antimicrobial elements, and cytokines. For liquid biopsy, saliva presents many advantages over blood, with its collection being easy, safe, non-invasive, and cost-effective. Salivary diagnostics have disclosed the existence of several molecular indicators of local and systemic disorders, including cancer [7,8].

Interest in salivary miRNAs as novel non-invasive tools for the diagnosis of cancer and systemic disease has rapidly increased.

The present systematic review has been designed in order to answer the question “Are salivary microRNAs reliable biomarkers for diagnosis of cancer and systemic diseases?”, formulated according to the “Population or problem”, “Intervention or exposure”, “Comparison”, “Outcome” (PICO) worksheet [9].

## 2. Results

**Figure 1:**
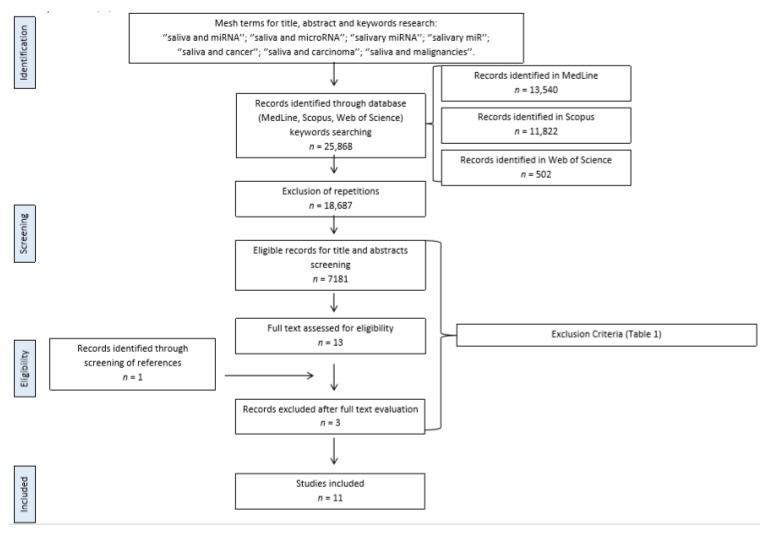
Flow-chart diagram for the selection of 11 papers included in the review.

Search flow is summarized in Figure 1. Multiple databases were searched for titles, abstracts, and keywords returned 25,868 records; after exclusion of repetitions, 7181 records were screened for titles and abstracts (see inclusion and exclusion criteria, specified in Table 1).

Thirteen records were eligible for full-text reading. Three records were discarded at this level (Figure 1). 

Cross-reference checks returned one more record which was included in the full-text evaluation phase.

Eleven papers eventually fulfilled the inclusion and exclusion criteria. Table 2 shows the selected papers and their general characteristics such as title, authors, year of publication, disease investigated, selected biomarkers, analytical techniques used, and type of saliva collected [10,11,12,13,14,15,16,17,18,19,20].

### 2.1. Quality Assessment

**Figure 2:**
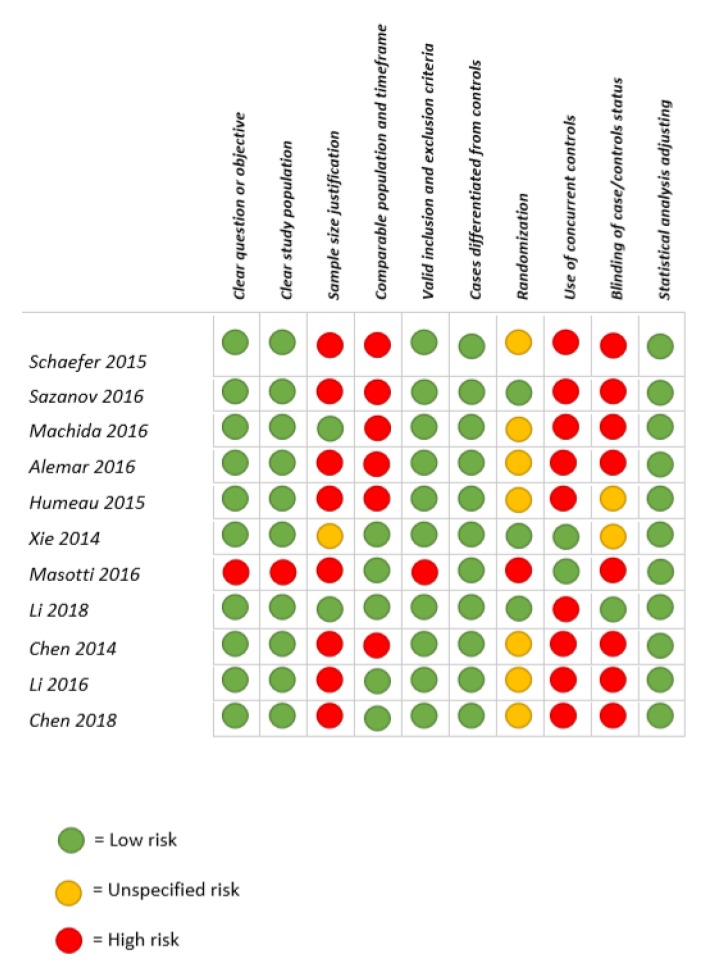
Critical appraisal, including main potential risk of bias and quality score.

A critical appraisal of the selected papers is reported in Figure 2.

Disagreement between reviewers occurred in four cases (47%) and all were resolved by discussion between the two independent assessors as follows:

(a) Paper 1 [10]: divergences involved National Institute of Health (NIH) question number 5, and after discussion, the study was scored as “intermediate”; (b) paper 5 [14] was scored as “intermediate” by one reviewer and “poor” by the other. Divergences about the use of statistical analysis were discussed and the paper was scored as “intermediate”; (c) paper 6 [15] was scored as “good” by one reviewer and “intermediate” by the other. After discussion, the article was scored as “good”; (d) paper 7 [16] was scored as “poor” and “intermediate” by one and the other assessor, respectively. After discussion, both reviewers chose to score the study as “poor”.

The most frequently encountered risks of bias (ROBs) were the absence of data regarding the status of participants (e.g., disease/healthy) to saliva investigators (blind procedure) (10 studies; 91%), the lack of sample size justification (nine studies; 82%), the absence of concurrent controls (eight studies; 73%), and the lack of randomization (eight studies; 73%). Less frequent ROBs were the absence of a comparable population for recruitment and correspondence of timeframe of recruitment (six studies; 54,5%), non-appropriate study question and objectives, in addition to a non-well-defined study population, and to the choice of non-valid and unreliable inclusion and exclusion criteria (one study).

Two “good” quality researches were identified among the group of the 11 selected studies (18.2%; studies 6 and 8). Eight records (73%; studies 1, 2, 3, 4, 5, 9, 10, and 11) were classified as “intermediate”, and one (9%; study 7) was classified as having “poor” quality.

### 2.2. Level of Evidence

Application of the Oxford Center for Evidence-based Medicine (CEMB) guidelines highlighted that all of the selected papers have a low level of evidence (4 on 5, the fifth level being the lowest), because of their case-control or case series design.

### 2.3. Disease Investigated

Two studies (1 and 2) were focused on colorectal cancer (CRC) and inflammatory bowel diseases (IBD). Crohn’s disease (CD) was compared with ulcerative colitis (UC) to assess the expression of salivary miRNA target. Distal adenocarcinoma, an anatomical variant of CRC, was studied in paper 2 to compare miRNAs in the serum and saliva of cancer patients with those of healthy controls (HC) [10,11].

Four studies investigated pancreatic oncological diseases. Specifically, paper 3 investigated the presence of two specific miRNAs in salivary exosomes, through a comparison between pancreatic-biliary tract cancer patients and HC [12].

Serum and saliva from pancreatic ductal adenocarcinoma (PDAC) patients were compared to HC specimens in study 4: four specific salivary miRNAs targets were investigated [13].

Study 5 was focused on pancreatic cancer (PC); salivary miRNA expression was compared between patients with PC, pancreatitis, inflammatory conditions, and HC [14]. 

The case-control study 6 investigated circulating miRNAs for detecting resectable PC, comparing salivary specimens of PC patients and HC [15]. 

The relationship between PC and ZHENG (a concept of Traditional Chinese Medicine—TCM) was studied in paper 9. The expression of salivary miRNAs was investigated in a case-control setting [18].

In article 7, salivary and blood miRNA detectability was explored in patients with fibromyalgia (FM) [16].

Study 8 was conducted to investigate if salivary miRNAs were reliable biomarkers for the diagnosis of gastric cancer (GC) [17].

Study 10 investigated the pathogenesis of gastric ulcer in association to *Helicobacter pylori* by identification of selected miRNAs in saliva, serum, and gastric tissues of cases and controls [19].

Study 11 explored salivary miRNA expression in subjects of yang or yin deficiency constitution and in subjects with balanced constitution, a concept of TCM. Subclassification of patients into these group was performed through the use of Wang Qi’s Body Constitution Classification Questionnaire—Chinese, version ZYYXH/T 157-2009.

Salivary analysis from patients with one or the other constitution category and HC allowed the identification of specific miRNAs [20].

### 2.4. Type of Saliva and Analytical Techniques

Data on type of saliva, methods and procedures of collection, handling, storing, and analytical techniques are reported in Table 3.

Salivary miRNA identification was conducted onto whole saliva samples in each study.

Particularly, the authors of studies 1, 3, 4, 5, 8, and 11 collected unstimulated saliva, whereas studies 6 and 10 reported the sampling of saliva through stimulation with citric acid solution. In three papers (2, 7, and 9), no information on such an aspect was provided.

Data regarding precise instructions given to patients before saliva collection were available for eight studies. In six of these (2, 4, 6, 8, 10, and 11), subjects were asked not to eat and drink for a period of time ranging from 30 min to 2 h before saliva sampling. Patients in study 8 were allowed to drink water. Additionally, subjects in studies 4, 5, 6, 10, and 11 did not perform oral hygiene procedures before collection. Patients in studies 4, 6, 10, and 11 were also asked to refrain from smoking. Saliva was taken in the morning in the three studies (2, 3, and 4) where this information was available. 

Most of the studies (8 out of 11; Table 3) reported more or less specific data on handling (including centrifugation) and storing of saliva. Particularly, centrifugation methods were rather heterogenous, the most frequently used values ranged between 2500–3000× *g* for 10 to 15 min at 4 °C. It seems worthy to mention here that supernatants in studies 6 and 9 underwent a second cycle of centrifugation at 12,000× *g* for 10 min and at 10,000× *g* for 1 min, respectively. Studies 1, 4, 5, and 8 also reported data on RNA stabilization.

The most frequently reported method of sample storing was freezing at −80 °C (studies 3, 5, 6, 8, and 9).

Qualitative and quantitative miRNA expression was investigated by Reverse Transcription real-time qualitative Polymerase Chain Reaction (RT-qPCR) in 10 studies. One research was performed through stand-alone miRNA micro-arrays (study 11). Two studies associated micro-arrays to qPCR for miRNAs detection (studies 1 and 10).

## 3. Discussion

Salivary analysis is transforming the concept of non-invasive diagnosis, providing novel options in the panorama of the so-called “liquid biopsy”.

Evidence exists that several salivary biomarkers (including genetics, transcription molecules, proteins, metabolites, and lipids) can accurately describe peculiar pathological and physiological states [21]. Examples of diseases that can reliably be diagnosed through salivary analysis are pancreatic (miR-3679-5p and miR-940) [15], lung (cytokines IL1RN, IL1B, CXCL10) [22], and breast cancers (phenylalanine, tryptophan) [23], as well as myocardial infarction (C-reactive protein, myoglobin, and myeloperoxidase) [24].

Among salivary biomarkers, miRNAs seem very promising, both for early diagnosis and for understanding the pathogenesis of some diseases (e.g., oral cancer, salivary glands cancer, neurological or psychiatric deficiencies) [25]. Moreover, it has been demonstrated that salivary transcriptome is very abundant, consisting of thousands of mRNAs and miRNAs [26,27].

Despite the demonstration that the presence of an inflammatory or malignant lesion in a site distant from the oral cavity is related to peculiar miRNAs in the salivary fluid, such an occurrence is still poorly understood [28]. It has been speculated that the expression of some miRNAs in saliva could be very similar to those in serum.

There are several hypotheses trying to explain the detection of cell-free nucleic acids (including miRNAs) in saliva. It has been reported that blood-derived molecules—entering salivary gland tissue via various cellular mechanisms, such as transcellular (passive intracellular diffusion and active transport) or para-cellular routes (extracellular ultrafiltration)—affect the molecular composition of oral fluids. miRNAs could also be produced locally by apoptosis or cell necrosis, and they could also be released by normal epithelial or cancerous cells in exosomes or micro vesicles [29,30].

The present systematic review has highlighted a rapidly increasing interest toward the use of salivary miRNAs for diagnosis of systemic diseases and cancers. Nevertheless, applying strict inclusion and exclusion criteria for selecting only papers reporting sufficient diagnostic and biochemical information, the number of studies is limited to 11. 

As shown in Table 4, most researches (7 out of 11) showed statistically significant correlations between one or more miRNAs and the investigated disease. Even though such results would, in general, indicate that specific salivary miRNAs can contribute to the diagnosis of diseases, our critical appraisal highlighted that most of the included studies did not satisfy a relevant percentage of items suggested by the NIH format. Particularly, only two studies received a score indicating “good” quality [18,20]. Furthermore, the level of evidence of all of the examined studies appears quite low, due to their case-control design.

ROBs most frequently accounting for decreased quality of the study are the absence of information on the status of participants (e.g., disease/healthy) to saliva biochemical analysts (blind procedure) (10 studies; 91%) and the lack of sample size justification (nine studies; 82%).

It is worthy to mention here that one of the studies considered as being of good quality did not report a sample size justification (study 6). The other (study 8) was conducted following the principles of the prospective-specimen-collection, retrospective-blinded-evaluation (PRoBE) guidelines, and therefore, even if not clearly reported, it is likely that the authors calculated a sample size [31]. 

Blinding the status of participants (e.g., the use of anonymous labels) to specimen evaluators is of paramount importance in researches attempting to identify biomarkers. The absence of a blinding procedure increases the probability of subjective interpretation of results, thus leading to misinterpretation of biomarker importance.

The use of untargeted procedures for establishing a relationship between salivary miRNAs and a specific pathology usually leads to the identification of a large number of molecules. Among these, only few, if any, will be validated as biomarkers. The process leading to the identification of one reliable miRNA among the whole group of detected molecules requires a statistically adequate number of cases. Nevertheless, only a few will be able to reliably represent a pathological state, based on their statistical significance.

The sample size justification is connected to the sensitivity and specificity of the potential biomarker. Depending on the number of considered variables, a statistically driven population size selection must be performed during the design stage; the unbalancing of cases vs. healthy controls could give rise to confounding results during the selection, verification, and validation stages.

Whole saliva is a mixture of fluids produced by parotid, submandibular, sublingual and minor salivary glands as well as by the gingival sulcus (crevicular fluid). It is well known that all such biological liquids vary with regard to their chemico-physiscal properties [32]. Apart from molecules directly deriving from the body (either from the oral cavity or from distant sites), saliva contains a myriad of substances produced by microorganisms and of exogenous origin (e.g., food-derived components). Moreover, saliva quantity and quality from the same subject may be grossly influenced by factors such as age, gender, circadian rhythm, diet, drugs and environmental exposures [32].

All of the studies included in the present review took into consideration whole saliva specimens. Apart from the mentioned ROBs, on the basis of the complexity of such fluid, it is crucial to critically evaluate results, considering variables related to methods and procedures of collection as well as to handling and storing of samples. Aspects such as oral hygiene sessions and/or food or drink intake in proximity to the collection of specimens can affect saliva quality and interfere with the identification of target molecules [33].

Both of the studies considered as being of good quality (6 and 8) reported precise data on instructions given to patients before saliva collection. However, while study 8 reports a time period of 2 h, patients of study 6 were asked to refrain from eating and drinking (except for water) for only 30 min before sampling. The variability of procedures adopted demonstrates, to some extent, the lack of agreement and standardization among groups of researchers and the undoubtful need for further research in such a specific field.

Centrifugation of specimens is another key factor that may influence transcripts identification, as contamination may alter microRNA profiles. Both the studies labeled as “good” (6 and 8) report data on centrifugation, even though they are not comparable. The procedures involving two separate cycles of centrifugation (studies 6 and 9) seem to be more reliable to obtain acceptable samples for miRNAs evaluation. Almost half of the studies (5 out of 11) did not report data on centrifugation and all of these belong to the “intermediate” or “poor” quality categories. As with what was reported for procedures of collection, also handling of saliva for miRNA analysis requires more research altogether with the definition of standardized protocols.

### 3.1. Pancreatic Cancer

The most investigated disease in the present review was pancreatic cancer (five studies). Such a tumor is the seventh leading cause of cancer-related deaths worldwide. Because patients rarely exhibit symptoms until an advanced stage of the disease, pancreatic carcinoma remains one of the most undiagnosed and lethal malignant neoplasms. Globally, 458,918 new cases were reported in 2018. Despite advancements in the detection and management of PC, the 5-year survival rate is only 9% [34]. 

Overall, 18 salivary miRNAs (miR-1246, miR-4644, miR-21, miR-34a, mir-155, miR-200b, miR-376a, miR-23a, miR-23b, miR-29c, miR-210, miR-216, miR-940, miR-3679-5p, miR-17, miR-18b, miR-18a, and miR-196a), detected through targeted and untargeted methods, have been studied in relation to the presence of pancreatic cancer, irrespective of the stage of development of the tumor [12,13,14,15,18]. 

miR-21 is one of the most investigated miRNAs in oncology (e.g., colorectal, thyroid, breast, ovarian, and cervix cancers) and it is known to inhibit the expression of phosphatases, thus limiting pathways such as AKT and MAPK [35,36]. This molecule appears to be constantly upregulated in PC and to be indicative of poor survival, response to treatment, and/or metastatic disease [37]. 

In the present review, three studies attempted to find a correlation between salivary miR-21 and PC [13,14,18], with controversial results. Study 4 demonstrated that miR-21 is overexpressed in patients with PC, even though the results are not statistically significant and therefore the molecule cannot be used to distinguish patients from healthy controls [13]. On the other hand, studies 5 and 9 report significant results regarding salivary miR-21 sensitivity and specificity for identifying patients with PDAC [14,18]. All of these three studies have been classified has having “intermediate” quality.

Apart from patients with PC, miR-21 has been investigated also on patients with CRC and IBD, within studies considered in the present review (studies 1 and 2). According to our critical appraisal, the three studies are not labelled as having “good” quality. Nevertheless, it seems worthy to mention here that the molecule results overexpressed in all the categories of patients. Therefore, such a miRNA does not seem to be very useful for indicating the presence of a specific disease. The same observation can be done for miR-23a, which is overexpressed in PC and in fibromyalgia patients.

Study 9 also reported on the association between salivary miRNAs in patients with PC belonging to three different types of ZHENG. ZHENG is the cornerstone in TCM theories. In cancer occurrence and its progression, a series of ZHENGs are involved, potentially influencing treatment. According to the study, miR-17, miR-21, miR-181b, and miR-196 are differently expressed in patients with PC and with Shi-Re ZHENG, Pi-Xu ZHENG, and Xue-Yu ZHENG [18]. The authors hypothesize that such differences could give some suggestions about three different pathogeneses of PC. However, it seems important to highlight here that the paper does report precise hypotheses on pathogenesis, as well as on criteria for ZHENG differentiation.

miR-1246 and miR-4644 are supposedly involved in tumor initiation, establishment, and metastatic process [38]. Particularly, salivary expression of miR-1246 is significantly related to serum levels of the well-known protein CA19-9 [39,40,41]. It has been speculated that concentration of such miRNA in saliva is directly associated with concentration of the same molecule in PC tissue [42].

When salivary miR-1246 and miR-4644 are simultaneously taken into consideration, sensitivity and specificity for PC diagnosis are increased with respect to biomarkers analyzed singularly [13].

miR-940 regulates the 3′-UTR of the GSK3β and sFRP1 genes’ expression, resulting in Wnt/β-catenin signaling activation. Upregulation of miR-940 suppresses GSK3β and sFRP1 and could promote pancreatic carcinoma proliferation and invasion [43]. miR-3679-5p seems to activate the cytoskeleton regulator RNA (CYTOR), which plays a pivotal role in the development and progression of a variety of cancers [44]. 

According to the results of study 6, combined analysis of salivary miR-940 and miR-3679-5p has better discriminatory power than CA19-19 in detecting resectable PC with good sensibility (90% and 83% for miR-940 and miR-3679-5p, respectively) and acceptable sensitivity (40% and 45%, respectively) [15]. The positive predictive value of the model combining the two miRNAs was 78.9%, and the negative predictive value was 76.2%. Furthermore, Study 6 was labeled as a “good” quality study after critical appraisal. It seems therefore advisable to highlight here the potential important role of salivary miR-940 and miR-3679-5p as reliable markers for PC. 

Salivary miR-23a-3p and miR-23b-3p were overexpressed and capable of distinguishing PC patients from HC and pancreatitis (study 5 [14]) with appreciable sensitivity and specificity; considering the regulatory role in pancreatic inflammation processes, Grieco et al. [45] described how these miRNAs were found to regulate the expression of the pro-apoptotic Bcl-2 proteins DP5 and PUMA and consequent human β-cell apoptosis. These results identify a novel cross-talk between a key family of miRNAs and pro-apoptotic Bcl-2 proteins in human pancreatic β-cells, broadening our understanding of cytokine-induced β-cell apoptosis in early type 1 diabetes.

### 3.2. Gastric Cancer and Gastric Ulcer

According to the GLOBOCAN 2018 data, gastric cancer is the fifth most common neoplasm and the fifth most deadly cancer, with an estimated 783,000 deaths in 2018 [46].

Among studies of the present review dealing with GC, study 8 was labeled as having “good” quality [17]. The research reports the analysis of differences in salivary transcriptomics, including several miRNAs, between a cohort of patients with GC and controls. Particularly, six salivary miRNAs (miR-140-5p, miR-301a, miR-374a, miR-454, miR-15b, and miR-28-5p) were found to be significantly downregulated (*p* < 0.05) in patients without GC, yielding an Area Under Curve values from 0.63 to 0.70, thus being validated as candidate biomarkers. Among these, only two (miR140-5p and miR-301a) yielded an AUC of 0.81, satisfying a prediction model for diagnosis of GC.

Conversely, it has been demonstrated that miR-140-5p expression is decreased in specimens of GC, when compared to healthy mucosal tissue [45]. miR-140-5p is able to suppress the proliferation, migration, and invasion of GC by directly targeting the 3′-UTR of YES1 [47]. 

The possible discrepancy between extracellular salivary and tissue miR-140-5p seems to deserve more investigation.

miR-301a is an oncogenic miRNA playing an important role in activating tumor cell invasion/migration, promoting cell proliferation, inhibiting apoptosis, and enhancing chemosensitivity, both in vivo and in vitro [48]. With specific regard to GC, it has been shown that the abnormal expression of miR-301a is associated with cancer progression and poor prognosis [49].

GC incidence and mortality are highly variable by region and highly dependent on diet and *Helicobacter pylori* infection [50]. The relationship between infection and expression of salivary miRNA in relationship to MMP-9 in patient with *Helicobacter pylori*-associated gastric ulcer was investigated in study 10 (“intermediate” quality) [19]. Real-time PCR analysis showed that miR-204 was significantly downregulated in ulcer tissue, blood, and saliva samples from *Helicobacter pylori*-associated gastritis patients compared with healthy controls. These results suggest that miR-204 may contribute to the regulation of *Helicobacter pylori*-associated gastritis by targeting MMP-9 mRNA.

Many studies have revealed that miR-204 plays a role as a tumor suppressor [51]. Regarding gastric cancer, validated target genes of miR-204 are BCL-2 [52], EZRIN [53], rabb22a [54], sirt1 [55], sox4 [56], and usp47 [54].

### 3.3. Colorectal Diseases

Two studies in the present review were focused on three colorectal diseases (Chron’s disease, ulcerative colitis, and colorectal cancer) [10,11].

Both studies were judged as having “intermediate” quality. 

UC induces diffuse inflammation of the colonic mucosa. CD results in the transmural ulceration of any portion of the gastrointestinal tract—most often the terminal ileum and colon. Patients affected by these inflammatory bowel diseases experience symptoms, including diarrhea, abdominal pain, bloody stools, and vomiting [57,58].

New cases of CD and UC diagnosed each year are approximately 11 and 12 per 100,000 population, respectively; median age at diagnosis for CD is 34.9 years, while it is 29.5 for UC [59].

The authors of study 1 [10] identified five salivary miRNAs (miR-21, miR-31, miR142-3p, miR-26a, and miR-101) in IBD patients. However, all such biomarkers failed to show statistical significance after applying Bonferroni correction for multiple testing [10].

miR-21 was more present in saliva of patients with UC than in HC. Apart from the well-known role in tumor promotion and development, miR-21 is also involved in the deregulation of pathways such as “platelet activation signaling and aggregation”. Platelets emerge as key players in the inflammatory cascade. The roles of platelets in platelet-mediated inflammation in CD patients and the critical role of miRNAs in the control of the mechanism of hemostasis may, in part, suggest that both inflammation and coagulation impairment could contribute to pathogenesis of UC [60]. 

As for miR-21, salivary levels of miR-31 were found to be overexpressed in UC patients compared to HC. miR-31 is implicated in several inflammation-associated disorders; its expression signature could differentiate CD, UC, microscopic colitis, and pediatric IBD. MiR-31 is a major regulator of Wnt, BMP, and TGF-β signaling, controlling intestinal stem cell proliferation, intestinal homeostasis, and colorectal cancer progression by binding the 3′-UTR of ERK5, RAS1, TGF-β, β-catenin, and several other mRNAs to control cell migration, cell proliferation in vitro, and tumorigenesis and metastasis in vivo [61].

Salivary miR-101 was found to be overexpressed in patients with CD. It has been hypothesized that miR-101 could restrain the migratory potential of cells by repressing the enhancer of zeste homolog 2 (EZH2) [62]; in fact, in vitro and in vivo studies showed how this peculiar miRNA is decreased in colon cancer tissues compared with adjacent non-tumor tissues [63]. Moreover, the overexpression of miR-101 suppresses cell proliferation and inhibits cell migration and invasion in HT-29 and RKO colon cancer cell lines [64].

Despite the scarce importance in IBD differentiation, the oncogenic role of miR-142-3p is well known, which promotes cellular invasion in colorectal cancer cells by activating RAC1 and the onco-suppressive activity of miR-26a, and inhibits cell aggressiveness by regulating FUT4 in CRC [65,66].

Study 2 [11] demonstrated the overexpression of salivary miR-21 among patients with CRC. The biomarker was tested both for CRC identification and for the discrimination of the stage of disease.

Despite the small sample size, analysis of salivary miR-21 returned a 91% specificity and 97% sensitivity (*p* = 5 × 10^−12^) distinguishing CRC from HC. However, the study was not able to provide a statistically significant distinction between cancer stages.

### 3.4. Others (Fibromyalgia, Gastritis, Traditional Chinese Medicine)

Fibromyalgia is a somewhat controversial condition characterized by chronic widespread pain, unrefreshing sleep, physical exhaustion, and cognitive difficulties. It occurs in all populations throughout the world, with an overall prevalence between 2% and 4% [67].

In article 7 of the present review [16], salivary and blood miRNA detectability was explored in order to study FM by the comparison of serum miRNAs in 14 cases and 14 controls (all females, matched for age). Six serum miRNAs (miR-23a-3p, miR-1, miR-133a, miR-346, miR-139-5p, and miR-320b) resulted significantly deregulated. Particularly, among patients with FM, miR-1 showed the greatest downregulation, followed by miR-23a-3p. Six statistically dysregulated target biomarkers were selected for the salivary investigation. Interestingly, only three miRNAs were detectable both in serum and saliva (miR-23a-3p, miR-346, and miR-320b). However, though being detectable, salivary miRNA expression values were not statistically different if compared to controls [19].

The biological effects dependent on genes targeted by dysregulated miRNAs in FM patients are, to different levels, involved in muscular atrophy, epilepsy and autism (miR23a-3p), myoblast differentiation and modulation of brain-derived neurotrophic factor (BDNF) expression (miR-1); myoblast differentiation (miR-133a), immune response (miR-346), brain development (miR-139-5p), neuron development, autism, and complex regional pain syndrome (miR-320p) [16].

In the context of TCM, study 11 explored salivary miRNAs expression in subjects of Yang or Yin deficiency constitution, as well as in subjects with a balanced constitution [20].

Yang deficiency and Yin deficiency constitution accounts for 9.0% and 8.3% of the distribution of nine constitutions of the TCM, respectively [68]. These unbalanced constitutions are considered to be related to diseases or are the risk factors of diseases. According to the authors, the research on TCM constitution has important clinical significance in the prediction and prevention of diseases [20]. Therefore, trying to find a relationship between the expression of salivary miRNAs and TCM constitutions, they attempted to establish a direct correlation between specific salivary molecules and the prediction of development of certain diseases. Such an association, however, seems, to some extent, somewhat artificial and not evidence-based, as none of the subjects included in Study 11 had specific diseases. Moreover, the criteria for identification of TCM constitutions are entirely based on self-reporting (questionnaire) and not on objective findings.

Differences in expression were highlighted for miR-4443 and miR-2681-3p in Yang deficiency constitution (main characteristic: cold intolerance) and for miR-4455 and miR-1343-3p in Yin deficiency constitution (main characteristic: heat intolerance). A role in thermoregulation is played by thyroid hormones T3 and T4 (triiodothyronine and thyroxine). In 2017, Yicheng et al. described how T CD4+ cells play a significant role in the pathogenesis of Graves’ disease, a common immuno-mediate condition; lymphocytic infiltration in thyroid gland leads to the production of autoantibody against thyroid-stimulating hormone receptor (TSH-r), which mimics the action of TSH, causing excessive thyroid hormone production and hyperthyroidism. miR-4443 can induce overexpression of cytokines, chemokines, and the proliferation of CD4+ T cells in vitro. The authors indicated that miR-4443 was elevated in GD patients and was significantly correlated with GD immune-pathogenesis [69]. 

Moreover, Shefler et al. recently reported that miR-4443 was present in micro-vesicles derived from activated T cells, which regulate mast cell activation by targeting PTPRJ gene, suggesting a role of miR-4443 as a regulator of inflammation [70].

The heterogeneity of the diseases investigated, as well as of salivary miRNAs, did not allow us to perform a meta-analysis of the studies included in the present review. Even when limiting the analysis to single groups of diseases (e.g., pancreatic cancer), researches report the experience of patients at different stages, therefore limiting the possibility of pooling data.

In conclusion, according to our critical appraisal, only two studies on salivary miRNAs have been performed following appropriate methodologies [15,17]. According to such studies, salivary miR-940 and miR-3679-5p seem to be reliable markers for pancreatic cancer, and miR140-5p and miR301a are promising molecules for the salivary diagnosis of gastric cancer.

Further studies, possibly avoiding the ROBs highlighted here, are necessary to consolidate these findings and to identify new reliable salivary biomarkers.

## 4. Materials and Methods

The Preferred Reporting Item for Systematic Reviews and Meta-analysis (PRISMA) statement was used to guide the present work [71].

### 4.1. Search Strategy

A multiple database research (Medline, Web of Science, Scopus) was set, using as entry terms: saliva and miRNA; saliva and microRNA; salivary miRNA; salivary miR; saliva and cancer; saliva and carcinoma; and saliva and malignancies. A periodic screening of the databases was performed between June 2018 and May 2019.

Only literature in English, published after 2000, was taken into consideration. Duplicates resulting from the initial database querying were discarded through End Note X9©, Clarivate Analytics, software aid.

First level screening was performed on titles, abstracts, and keywords by two independent investigators. In the case of controversial titles/abstract, full-texts were evaluated.

At the title/abstract screening level, case reports, conference proceedings, personal communications, letters to editor, and reviews were excluded.

Studies specifically reporting on biochemical methods, technological aspects, devices used, or proposed for saliva evaluation or detection of specific molecules were excluded.

The present systematic review takes into consideration only salivary miRNAs presumptively originating from tissues different from those in direct conjunction with the oral cavity. Therefore, we excluded studies evaluating miRNAs in the saliva of patients with pharyngeal, esophageal, salivary glands or oral cavity diseases, oral manifestations of systemic diseases, microbial infections, and drug/hormone dosages were excluded.

Studies considering miRNA expression in relationship to injuries/trauma and physical activity were not included. Furthermore, papers dealing with drug/hormone dosages were not considered. Moreover, papers investigating the association between salivary miRNAs and aging, psychiatric diseases, dementia, and cognitive evaluation were excluded. Studies on in vitro or animal models were also excluded. The use of salivary miRNAs for forensic purposes was not considered.

Inclusion and exclusion criteria are summarized in Table 1.

Literature eligible on the selected inclusion/exclusion criteria and reviews were double-checked, including reference lists, in order to identify papers possibly not considered in the previous selection.

Considering the study period and recruitment centers, a further assessment was performed to identify possible overlapping series of patients. Wherever multiple studies reported the same set of data in fully detectable series, only the most recent or the most complete series were included in the review.

### 4.2. Data Extraction, Quality Assessment, and Critical Appraisal

Information extracted from each study was summarized into an Excel table (Office Suite, Microsoft Corp.) which considered: title, authors, year of publication, disease investigated, number of involved patients, biomarkers (miRNAs), type of analytic device, type of saliva, and statistical significance.

Questionnaires issued by the National Heart, Lung, and Blood Institute within the National Institute of Health and called “Study Quality Assessment Tool” were used [72]. Such tools were specifically developed for each type of study (controlled intervention studies, systematic reviews with meta-analysis, observational cohort and cross-sectional studies, case-control studies, before–after studies without control group, case series studies) and they were answered by two independent investigators. Additionally, the number of patients enrolled in each study was taken into consideration.

Critical appraisal of studies has been summarized through assignation of a score ranging from 0% to 100%, based on the percentage of “yes” choices on the overall number of answers given. Studies having 80–100% score were labeled as “good”; those ranging from 50% to 70% were “intermediate”; and studies scoring less than 50% were defined as “poor”.

The level of evidence was assessed using the classification of the Oxford Center for Evidence-Based Medicine levels for diagnosis (2011) [73].

Disagreements were resolved by discussion between the reviewers.

## Figures and Tables

**Table 1:** Inclusion and exclusion criteria.

Inclusion Criteria	Exclusion Criteria
English literature Studies published between 2000 and 2019 Studies considering human saliva samples for miRNAs identification Studies using miRNAs as diagnostic biomarkers	Publication type: case reports, conference proceedings, personal communications, letters to editor, reviews Studies considering salivary miRNAs for diagnostic tools engineering, testing, and validation Studies on the diagnostic use of salivary miRNAs in patients with: • Pharyngeal, esophageal, and oral cavity diseases • oral manifestations of systemic disease • oral microbial infections • drug/hormone dosages • aging • psychiatric and cognitive diseases

**Table 2:** General characteristics of the selected papers.

Study Number	Title	Authors	Year of Publication	Disease Investigated	Number of Involved Patients	Biomarkers (miRNAs)
1 [10]	MicroRNA signatures differentiate Crohn’s disease from ulcerative colitis	Schaefer et al.	2015	CD vs. UC	35 HC, 42 CD, 41 UC, 5 saliva samples/group	miR-101
2 [11]	Plasma and saliva miR-21 expression in colorectal cancer patients	Sazanov et al.	2016	CRC	31 peripheral blood and 34 saliva samples (CRC stages II–IV) + 34 HC	miR-21
3 [12]	miR-1246 and miR-4644 in salivary exosome as potential biomarkers for pancreatic-biliary tract cancer	Machida et al.	2016	PC	12 PC and 13 HC	miR-1246 and miR-4644
4 [13]	miRNA-21 and miRNA-34a Are Potential Minimally Invasive Biomarkers for the Diagnosis of Pancreatic Ductal Adenocarcinoma	Alemar et al.	2016	PC	serum and saliva 24 PC + HC saliva, 10 PC, and 10 saliva HC	saliva: miR-21, -34a, -155, -200b, and -376a
5 [14]	Salivary MicroRNA in Pancreatic Cancer Patients	Humeau et al.	2015	PC	saliva from PC (7), pancreatitis (4), IPMN (2), and HC (4)	miR-21, miR-23a, miR-23b, and miR-29c
6 [15]	Salivary microRNAs Show Potential as a Non-invasive Biomarker for Detecting Resectable Pancreatic Cancer	Xie et al.	2014	PC	40 PC, 20 BPT, and 40 HC	miR-940 and miR-3679-5p
7 [16]	Circulating microRNA Profiles as Liquid Biopsies for the Characterization and Diagnosis of Fibromyalgia Syndrome	Masotti et al.	2016	FM	Blood and saliva, 14 FM, and 14 HC	miR-23a-3p, miR-346, and miR-320b
8 [17]	Discovery and Validation of Salivary Extracellular RNA Biomarkers for Non-invasive Detection of Gastric Cancer	Li et al.	2018	GC	Saliva, 63 GC, and 31 HC	miR140-5p, miR374a, miR454 4.61, miR15b, miR28–5p, and miR301a
9 [18]	MicroRNA Expression in Salivary Supernatant of Patients with Pancreatic Cancer and Its Relationship with ZHENG	Gao et al.	2014	PC and ZHENG	30 PC and 32 HC	5 miRNA candidates miR-17, miR-21, miR-181a, miR-181b, and miR-196a
10 [19]	Expression of miR-204 and MMP-9 in Helicobacter pylori-associated gastric ulcer	Li et al.	2016	*Helicobacter pylori*-associated GU	Ulcer, blood, and saliva samples of 46 patients with *H. pylori*-associated gastritis; normal stomach mucosa, blood, and saliva in 29 HC	miRNA-204
11 [20]	miRNA Expression Profile of Saliva in Subjects of Yang Deficiency Constitution and Yin Deficiency Constitution	Chen et al.	2018	Constitution deficit and miRNA	Saliva from 5 balanced individuals, 5 Yin deficiencies, and 5 Yang deficiencies	81 miRNA Yang deficiency 98 miRNA Yin deficiency

Legend: miRNA: micro RNA; CD: Crohn’s disease; UC: ulcerative colitis; HC: healthy controls; RT qPCR: reverse transcription quantitative polymerase chain reaction; CRC: colon-rectal cancer; PC: pancreatic cancer (including pancreatic-biliary tract cancer, pancreatic ductal adenocarcinoma); BPT: benign pancreatic tumor; IPMN: intraductal papillary mucinous neoplasm; FM: fibromyalgia; GU: gastric ulcer.

**Table 3:** Collection and analysis of saliva.

Study Number	Authors	Type of Saliva and Methods of Collection	Setting of Collection	Handling, Centrifugation, and Storing	Method of Analysis
1 [10]	Schaefer et al.	Whole Unstimulated	NR	Preservation: RNAprotect^®^ saliva reagent (Quiagen) Centrifugation: NR Storing: NR	RT-qPCR + microarray
2 [11]	Sazanov et al.	Whole NR	In the morning, empty stomach, after oral rinsing with sterile water	Preservation: NR Centrifugation: 12,000 rpm, 2 min Storing: NR	RT-qPCR
3 [12]	Machida et al.	Whole Unstimulated accumulation on the floor of the mouth and spit through a funnel	Collection between 7–12 am; Collection tube kept on ice at 4 °C	Preservation: NR Centrifugation: NR Storing: at 4 °C for 6 h, then at –80 °C	RT-qPCR
4 [13]	Alemar et al.	Whole Unstimulated	Collection between 9–11 am; No drinking, eating, smoking, or oral hygiene for at least 1 h before collection	Preservation: RNA Oragene^®^ RE-100 Centrifugation: NR Storing: NR	RT-qPCR
5 [14]	Humeau et al.	Whole Unstimulated, collected by micro-pipette	No toothbrushing 45 min before collection	Preservation: RNAprotect^®^ saliva reagent (Quiagen) Centrifugation: NR Storing: –80 °C	RT-qPCR
6 [15]	Xie et al.	Whole Continuous citric acid solution tongue stimulation for 5 s every 30 s	No eating, drinking, smoking, or oral hygiene procedures for at least 2 h before collection	Preservation: NR Centrifugation: 3 mL centrifuged (3000× *g*, 15 min, 4 °C) and re-centrifugation (12,000× *g*, 10 min, 4 °C) Storing: –80 °C	qPCR
7 [16]	Masotti et al.	Whole NR	NR	NR	qPCR
8 [17]	Li et al.	Whole Unstimulated	No eating or drinking except water for 30 min collection	Preservation: SUPERase-In RNase inhibitor^®^ Centrifugation: 5mL saliva (2600× *g* for 15 min at 4 °C). Storing: –80 °C	RT-qPCR
9 [18]	Gao et al.	whole NR	NR	Preservation: NR Centrifugation: saliva (2500× *g* for 10 min at 4 °C) and supernatant centrifuged (10,000× *g* for 1 min) Storing: −80 °C	RT-qPCR
10 [19]	Li et al.	whole Continuous 2% citric acid tongue stimulation	No eating, drinking, smoking, or oral hygiene procedures for at least 2 h before collection	NR	qPCR + microarray + ELISA
11 [20]	Chen et al.	whole Swabbing for 15 min	No eating, drinking, smoking, or oral hygiene procedures for at least 2 h before collection	NR	microarray

Legend: miRNA, micro RNA; NR, not reported; RT qPCR, reverse transcription quantitative polymerase chain reaction; CD, Crohn’s disease; UC, ulcerative colitis; CRC, colon-rectal cancer; PC, pancreatic cancer (including pancreatic-biliary tract cancer, pancreatic ductal adenocarcinoma); FM, fibromyalgia; GU, gastric ulcer.

**Table 4:** Statistical significance of salivary miRNAs in association with systemic disease and cancer.

Study	Disease Investigated and Location	Salivary miRNAs	Subjects	Status	Sensitivity	Specificity	Statistical Significance
1 [10]	Intestinal bowel disease	miR-101	CD vs. HC	overexpressed	NR	NR	*p* < 0.05
		miR-21, miR-31, miR-142-3p	UC vs. HC	overexpressed	NR	NR	*p* < 0.05
		miR-142-5p	UC vs. HC	underexpressed	NR	NR	*p* <0.05
2 [11]	Colon-rectal cancer	miR-21	CRC stages II/III/IV vs. HC	overexpressed	97%	91%	*p* = 5 × 10^−12^
3 [12]	Pancreatic cancer	miR-1246	PC vs. HC	overexpressed	66,70%	100%	*p* = 0.008
		miR-4644	PC vs. HC	overexpressed	75%	76,90%	*p* = 0.026
		miR-1246 + miR-4644	PC vs. HC	overexpressed	NR	NR	*p* = 0.005
4 [13]	Pancreatic cancer	miR-21	PC vs. HC	overexpressed	NR	NR	NR
		miR-34a, miR-155, miR-200b, miR-376a	PC vs. HC	NR	NR	NR	NR
5 [14]	Pancreatic cancer	miR-21	PC vs. HC	overexpressed	71%	100%	*p* = 0.012
		miR-23a	PC vs. HC and pre-cancerous	overexpressed	85,70%	100%	*p* = 0.001
		miR-23b	PC vs. HC and pre-cancerous	overexpressed	85,70%	100%	*p* = 0.014
		miR-29c	PC vs. HC	overexpressed	57%	100%	*p* = 0.03
		miR-210	Pancreatitis vs. HC	overexpressed	100%	100%	*p* = 0.000014
		let-7c	Pancreatitis vs. HC	overexpressed	75%	80%	*p* = 0.033
		miR-216	PC vs. Pancreatitis	overexpressed	50%	100%	*p* = 0.024
6 [15]	Pancreatic cancer	miR-940	PC vs. HC	overexpressed	90%	40%	*p* < 0.006
			PC vs. BPT	overexpressed	62,50%	75%	*p* < 0.004
			PC vs. HC + BPT	overexpressed	90%	41,70%	*p* < 0.001
		miR-3679-5p	PC vs. HC	underexpressed	83%	45%	*p* < 0.008
			PC vs. BPT	underexpressed	90%	45%	*p* < 0.007
			PC vs. HC + BPT	underexpressed	85%	45%	*p* < 0.002
7 [16]	Fibromyalgia	miR-23a-3p	FM vs. HC	NR	NR	NR	NR
		miR-346		NR	NR	NR	NR
		miR-320b		NR	NR	NR	NR
8 [17]	Gastric Cancer	miR140-5p	GC vs. HC	underexpressed	75%	83%	*p* < 0.05
		miR301a		underexpressed			
		miR374a		underexpressed	NR	NR	
		miR454		underexpressed	NR	NR	
		miR15b		underexpressed	NR	NR	
		miR28-5p		underexpressed	NR	NR	
9 [18]	Pancreatic Cancer/ZHENG	miR-17	PC vs. HC	NR	NR	NR	NR
		miR-21		NR	NR	NR	NR
		miR-181		NR	NR	NR	NR
		miR-196a		NR	NR	NR	NR
10 [19]	*Helicobacter pylori*-associated Gastric Ulcer	miRNR-204	GU vs. HC	NR	NR	NR	*p* < 0.01
11 [20]	Constitution deficit/yin yang	miR-4443	Yang deficiency constitution	underexpressed	NR	NR	NR
		miR-2681-3p		overexpressed	NR	NR	NR
		miR-4455	Yin deficiency constitution	underexpressed	NR	NR	NR
		miR-1343-3p		overexpressed	NR	NR	NR

Legend: NR, not reported; miRNA, micro RNA; CD, Crohn’s disease; UC, ulcerative colitis; HC, healthy controls; RT qPCR, reverse transcription quantitative polymerase chain reaction; CRC, colon-rectal cancer; PC, pancreatic cancer (including pancreatic-biliary tract cancer, pancreatic ductal adenocarcinoma); BPT, benign pancreatic tumor; IPMN, intraductal papillary mucinous neoplasm; FM, fibromyalgia; GU, gastric ulcer.
